# Report on Orthopedic Surgery, Made at the Annual Meeting of the Illinois State Medical Society, Convened in Chicago, May 3d, 1864

**Published:** 1864-12

**Authors:** David Prince

**Affiliations:** Jacksonville, Ill.


### CHICAGO MEDICAL JOURNAL.

VOL. XXI.] DECEMBER, 1864. [No. 12.

### ORIGINAL COMMUNICATIONS.

### REPORT ON ORTHOPEDIC SURGERY,

MADE AT THE ANNUAL MEETING OF THE ILLINOIS STATE MEDICAL SOCIETY, CONVENED IN CHICAGO, MAY 3D, 1864.

By DAVID PRINCE, M. D., Jacksonville, Ill.

(*Concluded from last month.*)

Mons. Bouvier is quoted by Barwell as having divided in 1842, in a dog, the flexor carpi radialis, the flexor carpi ulnaris, the flexor digitorum subliuris, and the flexor digitorum profundus. In none of them did the subcutaneous wound unite so as to restore the use of the parts. In another experiment, the tendons did not unite at all; in another, the severed structures even massed together. Mons. Bouley met with the last result in an experiment upon a horse.

It is probable that in some of these cases of massing together there would be afterward an absorption of portions of organized exudations, which impede the movements of tendons, like that which occurs after a general union of tissues in the neighborhood of fractures, so that the result finally would not be quite as bad as might be inferred from these statements.

An objection strongly urged even to the division of the tendo Achilles is, that the "cicatrix contraction" which attends all solutions of continuity united by the interposition of extensive organized exudation, gradually diminishes the distance between the cut extremities of the divided tendon, so that they are finally brought nearly or quite together. This makes a bad compensation for the advantage gained at first, by the necessity of the wearing of apparatus to prevent the recurrence of the deformity, while this process of cicatrix contraction is going on. In the treatment without tenotomy, the muscle is from the first made to grow longer, by a change of its nutrition, induced by the force gradually and persistently applied, rendering the progress at first more slow, while in the treatment by tenotomy, this growing of the muscle to a greater length has afterward to be secured, when the cure fallaciously seems to have been completed, and perhaps after the case has passed from under the supervision of the surgeon.

The following is Barwell's language upon this subject: "The re-union of the tendo Achilles, after its division for Talipes equinus, is almost a certainty, but it (the division) permanently weakens the muscles, nor is such a procedure, as a rule, an efficient cure of the disease; partly because the gastrocnemius and soleus are not the principal muscles affected, and generally have very little to do with the mal-position, partly because contraction is sure to recur." (p. 120.)

Notwithstanding all this, however, there are occasional instances in which even Mr. Barwell, anti-tenotomist as he is, would divide the tendo Achilles.

"I do not mean to deny that, occasionally, when there is either great want of development or great degeneration, it may be necessary to divide the tendo Achilles, but it should always be avoided if possible, since it is merely a temporary expedient which always leaves behind it a certain deformity." (p. 127.)

In contrast with this again is the language of Bauer. (p. 24.) "As a general thing you have only to deal with the contracted muscles, and *division* is the sovereign remedy. But if the case has existed from infancy the bones have in form accommodated themselves to their abnormal position, the tibio tarsal articulationis crippled; then the prognosis is rendered doubtful, and the case may be irremediable."

"It is a common observation of orthopedic surgeons that the relief of contracted muscles by tenotomy reacts most favorably for the nutrition of the affected extremity, and nutritive supply promotes, self-evidently, its growth and development. Passive motion co-operates in the same direction."

A question of interest here arises as to what part the division of the tendo Achilles takes in the restoration of the muscular function.

If the congestion of the muscle, occasioned by the increased supply of blood to the tendon beyond for the repair of its wound, favors a better nutrition and consequent restoration of nervous power, it might be supposed that a seton or an issue applied nearer to the muscle to be affected, from the proximity of inflammation, would do better by exciting more action in the muscles.

It is more probable that the movements of flexion and extension, which attend the treatment following the division of the tendons, and subsequent to the re-union of the divided tendons, gradually induces a lengthening of the muscular fibrils, and this lengthening is a necessary condition to their shortening under the irritation of electricity or any other irritant. This opposing force should be either elastic or alternating, in order to obtain the most stimulating effect upon the muscles in process of restoration; permitting frequent exercise of contraction, with yielding force so graduated as to restore the length of the muscle upon the decline or cessation of their contraction.

The alternating movements of the tendons of the still paralyzed antagonist muscles, first pushing and then pulling these tendons, and, in a minor degree, pushing and pulling the muscles themselves, invites a flow of blood to the muscular substance, favoring its continued healthy nutrition, and the earliest possible revival of nervous power, when the paralyzing cause residing in the brain, in the spinal cord, or in the course of the nerves, whether from organic lesion or sympathetic action, is removed.

If the cause of the paralysis is such a destruction of nervous substance as to result in complete and permanent paralysis, the alternating movements of the muscles will at least tend to preserve their volume, by keeping up their nutrition, by making it mechanically possible for the blood to circulate through all their capillaries; motion being as essential to the freest circulation through the muscles as through the lungs.

The general health then has the benefit of a well-distributed circulation, in addition to the local advantages of attention to this indication.

This plan of yielding force, called by Dr. Henry G. Davis "elastic extension," is very properly denominated by him, the "American plan," and to him is due the merit of having been the first to employ it systematically, and with a full appreciation of its value, acting in a manner similar to that of muscles, alternating in the extent of their movements with the alternations of the degrees of resistance to be overcome.

Apparently from ignorance of American Medical literature Barwell claims this plan as his own. This is one of the instances in which several claimants for originality may be equally honest and original, the merit, however, consisting in the application of some other invention which makes a revolution of the given art, not only easy but unavoidable.

In this case the invention at the bottom is the manufacture of elastic rubber, placing in every one's hands a most facile means of meeting an indication which the older surgeons saw but had no ready means of accomplishing. (See Trans. Am. Med. Assoc., 1863.)

In cases affording obstinate resistance to reduction by extension, the progress can be greatly facilitated by the occasional application of force, while the patient is insensible from the influence of ether.

The same condition is artificially produced which occurs in a subluxation or sprain. The most tense ligamentous fibres are torn, without a complete rupture. The investments of the muscular fibres in the shortened muscle are either slightly torn interstitially, or put upon extreme tension. All this is followed by increased vascularity, which is favorable to change of tissue, in obedience to the tension afterward applied to it for the purpose of elongation.

This has been a common practice among American Surgeons for many years, though Barwell, strangely enough, claims it as his peculiar invention. He says, with much apparent satisfaction, (p. 116,) "This is also a procedure of *my own* adaptation to these diseases, and is one from which very great advantage may be drawn." He very properly goes on to say, "I would limit its employment to severe cases, and would caution surgeons against the use of violence; since, when once the muscular power is annihilated by the anæsthetic, very little force is required to place the foot in a normal position."

ELECTRICITY.

Electricity has been employed to remove the condition of the muscles upon which the deformity has been supposed to depend.

This subject can not better be illustrated than by quoting from representative writers, who take opposite positions.

Bauer already so often quoted, more for the recent date of his publication than for its scientific value, says, "The most efficacious remedy in behalf of innervation is electricity. It should be used with assiduity every day and for months in continuation. It will stimulate the existing mobility and prevent structural decay. * * Electricity is the substitute for volition and the best local gymnastic agent. Next are friction with alcoholic liquids; with phosphorated oil, (phosphorus, 3 grs., dissolved in an ounce of warm almond oil,) with the flesh brush, with or without cold irrigation."

We are left to infer that he would apply the electric current to the contracted muscles with the intention of relieving the spasm upon which the contraction is supposed to depend. This question of spasm has been already sufficiently discussed and it may be proper to add that, as a curative agent, the galvanic current should not be applied to the muscles whose tendons it has been found necessary to divide, but to the elongated muscles whose partial or total paralysis has permitted the shortening of their antagonist muscles.

It is obvious that when, by unresisted tonic contraction, the muscular fibres and their fasciæ have shortened to their utmost, neither electricity nor the prick of a pin can make them shorten any more. A galvanic current can make no impression which is known by movements, because this agent and other irritants only produce contractions. If, however, the muscular fibrils and their investments are first made to grow longer by frequently repeated pulls upon them, or by constant force varying in intensity, thus restoring the muscle to a greater or less extent, to the possibility of performing its natural function, that of producing *motion*, instead of the one to which it had degenerated, that of holding parts in *position*, i. e., the function of ligaments, then, after so much progress has been made towards the cure, it might be expected that electricity would index it by the contractions which would result from its application.

It is difficult to see, however, on what rational principle, electricity should be applied to the shortened muscles with any other intention than to determine whether they could shorten any more, or to ascertain in the progress of treatment in a case in which a muscle had been shortened and degenerated beyond the possibility of exciting contractions by the electric current, whether any progress had been made, or perhaps to throw light upon the probable replacement of the muscular substance by fatty degeneration. In the latter case electricity could not produce movement.

The notion of Bauer that we have only to deal with the "contracted muscles," is certainly in forgetfulness of all correct pathology. He details in his book, cases of paralysis of the inferior extremity, followed by permanent extension of the foot, beginning with painless contraction of the extensor muscles. Now what would electricity do with these muscles? It might make them contract more disproportionately, or if in too strong currents it might exhaust their excitability. What would tenotomy do to them? It would permit a greater degree of shortening of the muscle affected than could otherwise take place. We have something else to deal with than the contracted muscles.

In these cases of paralysis of all the muscles of the leg, there was an attempt at restoration of muscular power, commencing in the triceps extensor pedis. The restored contraction of these muscles having no resistance to oppose followed the usual law of shortening and of acquiring a more limited space of contraction; or from utter want of pull upon them, a fixedness in the shortest space, to be followed by fatty degeneration, or by absorption of the proper substance of the muscles, and a condition of inelasticity in the muscular investments. In all such cases the early use of power to counteract the muscular contraction is an imperative indication; partly to obviate the permanent contraction of the muscles, which are in the process of restoration of their proper function, and partly to give time for restoration of contractility in the paralyzed antagonising muscles, which are slower in the process of restoration. If in the restoration of muscular contraction referred to in these cases of paraplegia, both sets of muscles had been supplied alike by nervous power, no deformity would have resulted. There remained a relative paralysis of the flexors of the foot, the tibialis anticus, peroneus tertius, and long extensors of the toes. If this is so, the electric current should be applied to the latter muscles, rather than to the calf of the legs.

The following quotation from R. B. Todd's "Clinical Lectures on Paralysis and Diseases of the Nervous System," Lindsay & Blakiston's Edit., p. 152, will here be in point:

"You will often be consulted as to some expedient for promoting the restoration of paralyzed limbs to their normal condition. To this question, after having given a fair trial to the various means which have been proposed for this purpose, I must reply that I know of nothing which more decidedly benefits paralyzed limbs than a regular system of exercise; active when the patient is capable of it, passive if otherwise.

"As to the use of electricity, which is now much in vogue, or strychnia, which has been recommended, I feel satisfied, as the result of a large experience, that the former requires to be used with much caution, and that the latter is apt to do mischief and never does good. I have seen cases in which, after the employment of electricity for some time, that agent has apparently brought on pain in the head, and has excited something like an inflammatory process in the brain. And so strychnia will also induce an analogous condition of the brain, *and will increase the rigidity* of the paralyzed muscles. Some good may occasionally be effected by the use of friction or cold water, or shampooing, all of which tend to improve the general nutrition of the nerves and muscles."

